# Yinchenhao Decoction Mitigates Cholestatic Liver Injury in Mice via Gut Microbiota Regulation and Activation of FXR-FGF15 Pathway

**DOI:** 10.3390/ph18070932

**Published:** 2025-06-20

**Authors:** Weiwei Li, Doudou Huang, Zichen Luo, Ting Zhou, Ziwen Jin

**Affiliations:** 1 School of Traditional Chinese Medicine, Shanghai University of Traditional Chinese Medicine, Shanghai 201203, China; 12024200@shutcm.edu.cn (T.Z.); jzw15088791427@126.com (Z.J.); 2 School of Pharmacy, Shanghai University of Traditional Chinese Medicine, Shanghai 201203, China; haungdoudou@shutcm.edu.cn; 3 Medical Metabolomics Center, Nanjing University of Chinese Medicine, Nanjing 210023, China; 20181398@njucm.edu.cn

**Keywords:** Yinchenhao decoction, gut microbiota, cholestasis, FXR-FGF15 signaling pathway, bile acid metabolism

## Abstract

**Objective:** Yinchenhao decoction (YCHD), a classical herbal formula comprising *Artemisia capillaris*, *Gardenia jasminoides*, and *Rheum palmatum*, has been clinically used for over 1000 years to treat cholestasis. However, its mechanism of action remains undefined. This study aimed to elucidate YCHD’s therapeutic mechanisms against cholestasis, with a focus on the gut microbiota-mediated regulation of the farnesoid X receptor (FXR)–fibroblast growth factor 15 (FGF15) pathway. **Methods:** An alpha-naphthyl isothiocyanate (ANIT)-induced cholestasis mouse model was established. Mice received YCHD (3/9 g/kg) for 7 days. 16S rRNA sequencing, targeted LC/MS (bile acid (BA) quantification), untargeted GC/MS (fecal metabolite detection), qPCR/Western blot (FXR pathway analysis), fecal microbiota transplantation (FMT), and antibiotic depletion were employed to dissect the gut–liver axis interactions. **Results:** YCHD alleviated cholestatic liver injury by reducing serum biomarkers, restoring BA homeostasis via FXR-FGF15 activation, and suppressing hepatic Cyp7a1-mediated BA synthesis. It remodeled gut microbiota, enriched FXR-activating secondary BAs (CDCA, DCA, CA), and restored the intestinal barrier integrity. Antibiotic cocktail abolished YCHD’s efficacy, while FMT from YCHD-treated mice enhanced its therapeutic effects, confirming microbiota dependency. **Conclusions:** YCHD mitigates cholestasis through gut microbiota-driven FXR activation and direct hepatobiliary regulation. These findings bridge traditional medicine and modern pharmacology, highlighting microbiome modulation as a therapeutic strategy for cholestatic liver diseases.

## 1. Introduction

Cholestasis is a medical condition marked by disruptions in bile production, secretion, and excretion, preventing the normal liver–intestine circulation. Its etiology is complex, with acute factors encompassing biliary obstruction, drugs, viral infections, etc.; chronic factors such as primary sclerosing cholangitis (PSC), biliary atresia, and primary biliary cholangitis (PBC); and inherited disorders like progressive familial intrahepatic cholestasis (PFIC) [1]. If not treated promptly, cholestasis can lead to liver fibrosis, ultimately leading to cirrhosis and eventual liver failure. At present, the U.S. Food and Drug Administration (FDA) has approved only two medications for managing cholestatic liver disease. The most widely recognized treatment is ursodeoxycholic acid (UDCA), a naturally occurring BA. The alternative, obeticholic acid (OCA), is prescribed for patients who either do not respond to or cannot tolerate UDCA. However, approximately 50% of patients do not respond to these two drugs [2,3]. The farnesoid X receptor (FXR), a BA-activated nuclear receptor, regulates BA homeostasis, inflammatory responses, and gut barrier integrity through its downstream effector FGF15/19 [4,5]. Despite their therapeutic promise, FXR agonists (e.g., GS-9674, EDP-305) and FGF19 analogs (e.g., NGM282) remain investigational and lack clinical approval [6,7,8]. The limitations of UDCA/OCA underscore the need for systems-level interventions. Targeting gut microbiota offers unique advantages: (1) It restores host–microbe co-metabolism of bile acids (BAs) and nutrients (e.g., rumen-protected lysine reshaping nitrogen flux [9]); (2) plant-derived therapies demonstrate clinical translatability, as seen in hyperuricemia where microbiota modulation reduces inflammation [10]; and (3) in fibrotic diseases like peritoneal dialysis-related fibrosis, microbial restructuring correlates with improved solute transport [11]. Thus, microbiota-centric strategies may overcome the bottleneck of single-target pharmacotherapy in cholestasis.

Mounting evidence confirms that gut microbiota dysbiosis intricately contributes to cholestatic pathogenesis [12,13,14]. Specifically, the intestinal tract—continuously exposed to external stimuli—generates metabolites, toxins, and hormones that translocate to the liver via the portal vein. While BAs serve as primary communicators of the gut–liver axis, in turn shaping gut microbiota composition, emerging research reveals broader microbial metabolic disruptions: the impaired production of microbiota-derived metabolites (e.g., SCFAs and phytosphingosine) compromises hepatic PPARα signaling, exacerbating inflammation and barrier dysfunction [15]. Dysbiosis triggers PARP-1-dependent parthanatos, a programmed cell death pathway linked to DNA damage and NLRP3 inflammasome activation [16]. Systemic effects occur via metabolite-driven post-translational modifications (e.g., butyrate-induced histone lactylation) influencing distal organs, as demonstrated in calcific valve disease [17]. Collectively, these mechanisms highlight gut microbiota as a central regulator of hepatobiliary and systemic homeostasis.

Yinchenhao decoction (YCHD), a classical herbal formula comprising *Artemisia capillaris*, *Gardenia jasminoides*, and *Rheum palmatum*, has been clinically used for over a millennium to treat cholestasis [18,19], with efficacy confirmed in clinical trials [20]. Critically, given that gut microbiota dysbiosis drives cholestatic pathogenesis via impaired FXR-FGF15 signaling, we hypothesize that YCHD alleviates cholestasis by remodeling microbial communities to activate the FXR-FGF15 axis. This premise is anchored in two key lines of evidence: (i) bioactive YCHD components (e.g., geniposide) directly activate the FXR-dependent BA regulation [21,22], and (ii) the intact formula confers hepatoprotection against toxins through microbiota modulation [23]. Notably, YCHD’s multi-target action aligns with broader gut microbiota-centric therapeutic paradigms: Microbial dysbiosis activates innate immunity (e.g., NOD2/NF-κB) in colitis-associated carcinogenesis [24]. Gut diversity governs host metabolic efficiency, as evidenced in livestock models [25]. Such mechanisms underpin interventions in sepsis and metabolic disorders where barrier protection and taxon-specific enrichment resolve inflammation. Thus, targeting gut microbiota represents a systems-level strategy to restore homeostasis.

To mechanistically validate the hypothesis that YCHD alleviates cholestasis through microbiota-driven FXR activation along the gut–liver axis, this study systematically investigates three interdependent questions: (1) whether gut microbiota is necessary for YCHD’s therapeutic efficacy; (2) how YCHD-remodeled microbiota reprograms BA metabolism to activate hepatic and intestinal FXR signaling; and (3) how microbiota-restructured FXR activation coordinates BA synthesis, transport, and barrier functions. This work aims to establish a causal chain linking microbial remodeling to host nuclear receptor pathways as the core mechanism underlying this traditional formula’s anti-cholestatic action.

## 2. Results

### 2.1. Chemical Characteristics of YCHD

**Figure 1:**
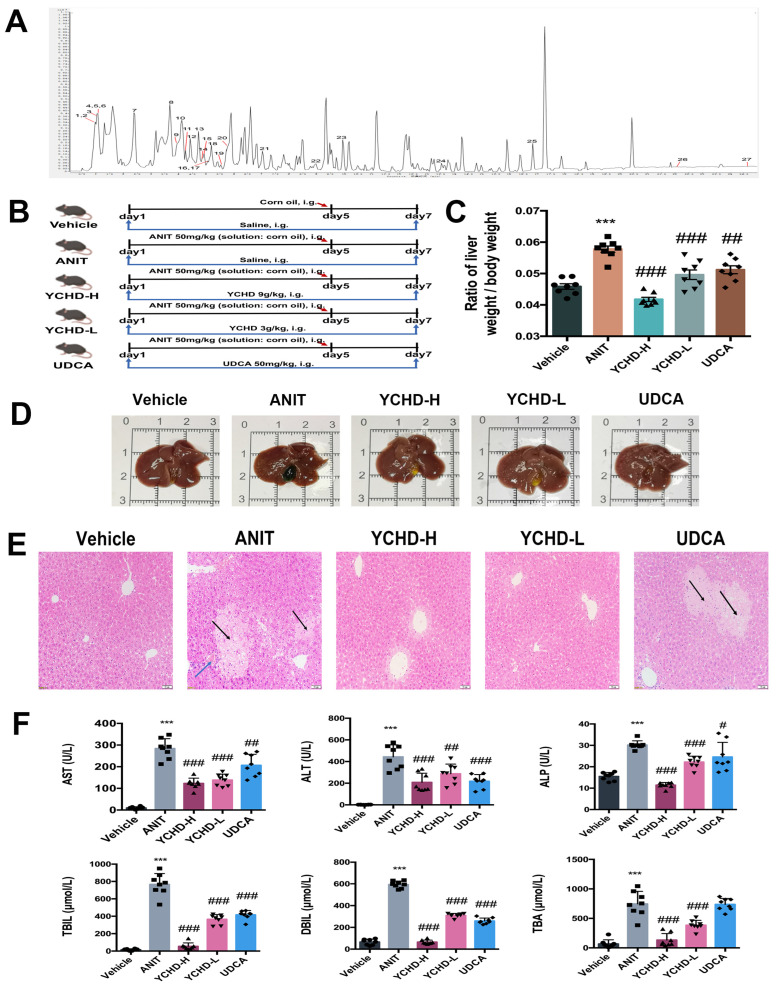
YCHD ameliorates cholestatic liver injury in ANIT-induced mice. (**A**) LC-MS chromatogram of YCHD in positive ion mode. (**B**) Experimental design schematic. (**C**) Liver-to-body weight ratios across groups. (**D**) Representative hepatobiliary morphology. (**E**) Hepatic histopathology (H&E staining). Scale bar: 50 μm. Black arrow indicates inflammatory infiltration of hepatocytes, and blue arrow indicates interlobular bile duct destruction. (**F**) Serum biomarkers (AST, ALT, ALP, TBIL, DBIL, TBA). All values are expressed as mean ± SEM. Compared with Vehicle group, *** *p* < 0.001; compared with ANIT group, # *p* < 0.05, ## *p* < 0.01, ### *p* < 0.001.

Using UPLC-Q-TOF/MS, 27 compounds were identified in YCHD (Table 1; Figure 1A). The categories of compounds included organic nitrogen compounds, carboxylic acids and their derivatives, flavonoids, phenolic compounds, ester compounds, glycosides, etc.

### 2.2. YCHD Ameliorates Cholestatic Liver Injury in ANIT-Induced Mice

To investigate YCHD’s therapeutic effects on cholestasis, we established an ANIT-induced murine model following the protocol in Figure 1B. ANIT challenge significantly increased liver-to-body weight ratios compared to controls. Both YCHD-treated groups (low/high-dose) and the UDCA group exhibited normalized ratios (Figure 1C). YCHD treatment effectively reduced ANIT-induced gallbladder enlargement and improved biliary obstruction (Figure 1D).

Histological evaluation through hematoxylin and eosin (H&E) staining revealed ANIT-induced hepatic inflammatory infiltration and bile duct damage (Figure 1E). YCHD administration significantly attenuated these pathological changes, particularly reducing the inflammatory area compared to the model group. Serum analysis confirmed YCHD’s hepatoprotective effects, with dose-dependent reductions in cholestasis biomarkers (ALT, AST, ALP, TBIL, DBIL, TBA) to near-normal levels (Figure 1F). These results demonstrate YCHD’s capacity to counteract ANIT-induced cholestatic liver injury through multiple pathological mechanisms.

Our findings also demonstrate that high-dose YCHD exhibits superior efficacy to UDCA in ameliorating liver-to-body weight ratio, histopathological parameters, and serum biochemical markers (Figure 1C,E,F). While UDCA remains the standard therapeutic agent for chronic cholestatic disorders like PBC, and its efficacy in this acute ANIT induction model is limited, which is consistent with previous mechanistic studies. Zhang et al. [26] systematically demonstrated that UDCA pretreatment (25–100 mg/kg) during the acute phase of ANIT-induced cholestasis paradoxically exacerbated hepatic injury, whereas therapeutic administration during the recovery phase effectively reduced cholestatic parameters. This phase-dependent therapeutic profile corroborates our observation that UDCA’s principal efficacy manifests during obstruction resolution rather than acute injury phases.

### 2.3. YCHD Enhanced BA Balance in Cholestatic Mice via FXR Pathway Modulation

**Figure 2:**
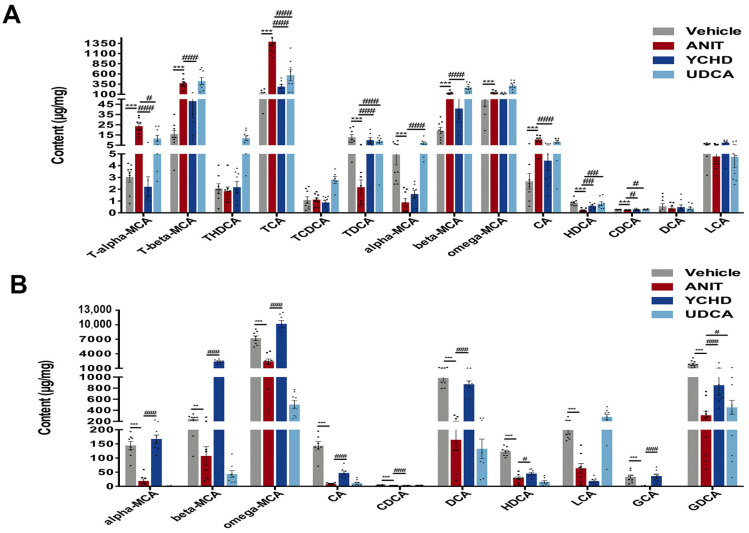
YCHD modulates hepatic and fecal BA homeostasis. (**A**) Hepatic BA profiles. (**B**) Fecal BA profiles. All values are expressed as mean ± SEM. Compared with Vehicle group, ** *p* < 0.01, *** *p* < 0.001; compared with ANIT group, # *p* < 0.05, ## *p* < 0.01, ### *p* < 0.001.

**Figure 3:**
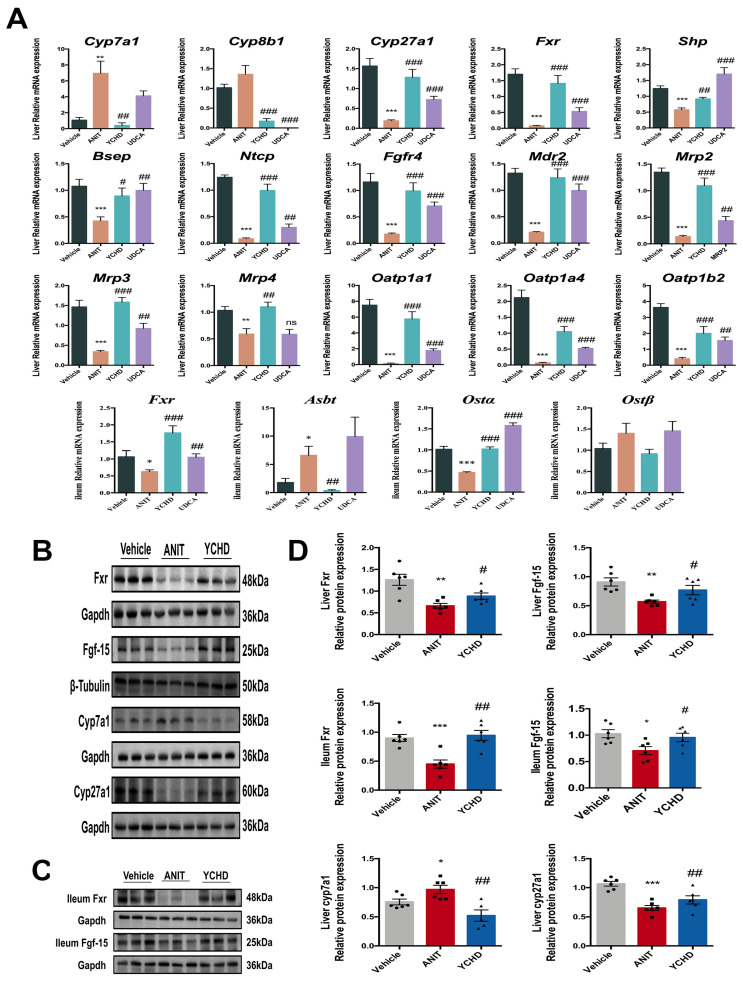
YCHD modulates BA homeostasis via FXR signaling. (**A**) Hepatic mRNA expression levels of Cyp7a1, Cyp8b1, Cyp27a1, Fxr, Shp, Bsep, Ntcp, Fgfr4, Mdr2, Mrp2, Mrp3, Mrp4, Oatp1a1, Oatp1a4, and Oatp1b2 and ileum mRNA expression levels of Fxr, Asbt, Osta, and Ostβ. (**B**–**D**) Western blot of Fxr, Ffg15, Cyp7a1, and Cyp27a1 in the liver and Fxr and Fgf15 in the ileum. All values are expressed as the mean ± SEM. Compared with Vehicle group, * *p* < 0.05, ** *p* < 0.01, *** *p* < 0.001; compared with ANIT group, # *p* < 0.05, ## *p* < 0.01, ### *p* < 0.001.

Cholestasis disrupts BA metabolism, prompting our investigation of YCHD’s regulatory effects using UPLC-MS/MS-based targeted metabolomics. Hepatic BA profiling revealed significant increases in rodent-specific muricholic acids (MCAs) including T-α-MCA, T-β-MCA, β-MCA, and ω-MCA in ANIT-treated mice versus controls, alongside elevated TCA and CA, while TDCA, α-MCA, HDCA, and CDCA showed marked reductions (Figure 2A). YCHD effectively normalized most ANIT-disrupted BAs, with the exception of ω-MCA and α-MCA. Mechanistically, the persistent elevation of ω-MCA and α-MCA reflects pathway-specific resistance: α-MCA synthesis occurs primarily via the alternative pathway (Cyp27a1/Cyp7b1-dependent), which showed partial response to YCHD (Cyp27a1↑ in Figure 3A). ω-MCA is derived from β-MCA through 6β-hydroxylation isomerization, a microbiota-dependent process impaired in cholestasis. Notably, MCAs are rodent-specific BAs with minimal human homologs (e.g., humans lack 6β-hydroxylase for ω-MCA synthesis). Their incomplete normalization highlights model-specific metabolic features with limited translational implications for human BA pools dominated by CA/CDCA.

Fecal BA analysis demonstrated parallel decreases in α-MCA, β-MCA, ω-MCA, CA, CDCA, DCA, HDCA, LCA, GCA, and GDCA in the ANIT group, consistent with impaired hepatobiliary BA excretion. YCHD treatment reversed these metabolic disturbances, upregulating all fecal BAs except LCA compared to the ANIT model (Figure 2B). This restoration indicates YCHD enhances BA efflux through the coordinated induction of hepatic transporters (Bsep, Mrp2) and intestinal reabsorption, thereby alleviating hepatic BA accumulation. Given the regulatory role of the FXR-FGF15 axis in BA homeostasis [27], we further analyzed tissue-specific FXR pathway activation. qPCR quantification demonstrated YCHD’s dose-dependent modulation of hepatic and ileal FXR signaling components, including the key BA synthesis enzymes and transporters (Figure 3). These molecular changes mechanistically explain YCHD’s capacity to reestablish BA homeostasis during cholestasis.

Our results demonstrated the significant suppression of the FXR-FGF15 axis in ANIT-induced cholestasis. Compared with controls, the ANIT group exhibited the reduced expression of Fxr (both mRNA and protein) and Fgf-15 protein in liver and ileum tissues, along with the decreased hepatic *Shp* and *Fgfr4* mRNA levels. YCHD treatment effectively reversed these changes, upregulating Fxr and Fgf15 expression compared to the ANIT model.

Hepatic BA synthase analysis revealed elevated Cyp7a1 and reduced Cyp27a1 expression (mRNA and protein) in the ANIT group. YCHD administration significantly decreased Cyp7a1 while increasing Cyp27a1 expression, promoting a metabolic shift toward alternative BA synthesis pathways. Critically, YCHD restored hepatobiliary transporter networks through the coordinated upregulation of canalicular exporters (Bsep, Mrp2) and sinusoidal exporters (Mrp3, Mrp4), while modulating ileal reabsorbers (Asbt, Ostα). These transporter alterations accelerated BA efflux independent of FXR signaling (Figure 3A,D, mechanistically explaining YCHD’s efficacy in reducing hepatic BA overload and improving cholestatic injury [28].

### 2.4. YCHD Alleviated Gut Microbiota Dysbiosis in Mice with Cholestasis

**Figure 4:**
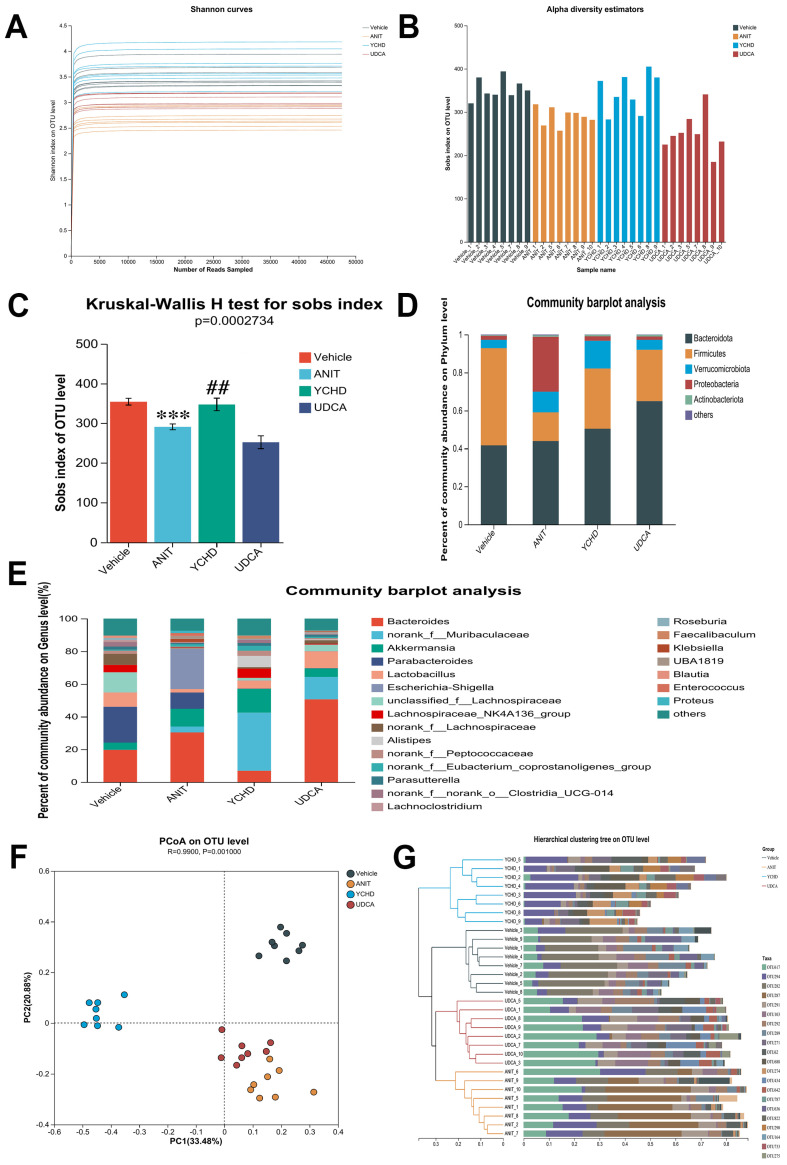
YCHD restores gut microbiota composition in cholestasis. (**A**) Shannon rarefaction curves. (**B**,**C**) α-Diversity indices (Sobs). (**D**,**E**) Phylum- and genus-level taxonomic profiles. (**F**) β-Diversity (PCoA). (**G**) Hierarchical clustering tree on OUT level. All values are expressed as mean ± SEM. Compared with Vehicle group, *** *p* < 0.001; compared with ANIT group, ## *p* < 0.01.

Given the gut microbiota’s critical involvement in BA homeostasis, we performed 16S rRNA sequencing to assess microbial changes. Microbial α-diversity, evaluated by the Shannon index, showed significant reduction in ANIT-induced cholestatic mice compared with Vehicle controls (Figure 4A–C). YCHD treatment partially restored microbial diversity parameters.

At the phylum level, the Vehicle group’s microbiota predominantly consisted of *Bacteroidota*, *Firmicutes*, *Verrucomicrobiota*, *Proteobacteria*, and *Actinobacteriota* (Figure 4D). ANIT challenge substantially altered this profile, exhibiting a notable decrease in *Firmicutes* abundance alongside increased *Proteobacteria* populations. YCHD intervention reversed these dysbiotic changes, elevating *Firmicutes* and reducing *Proteobacteria*. Genus-level taxonomic analysis further detailed the microbiota compositional shifts across experimental groups (Figure 4E).

Beta-diversity analysis through principal coordinate analysis (PCoA) revealed distinct microbial community structures across experimental groups (R = 0.99, *p* = 0.001; Figure 4F), with clear separation between YCHD-treated and ANIT-challenged mice. Hierarchical clustering further confirmed group-specific microbiota patterns, demonstrating significant compositional divergence (Figure 4G). Notably, the UDCA group clustered proximally to the ANIT model in both analyses, indicating minimal microbiota restoration despite comparable therapeutic efficacy. This spatial distribution pattern suggests YCHD’s anti-cholestatic effects involve microbiota remodeling, while UDCA’s mechanism appears mechanistically distinct from microbial regulation. The maintained β-diversity separation between YCHD and UDCA groups underscores their differential pathways for cholestasis management, despite shared clinical outcomes.

**Figure 5:**
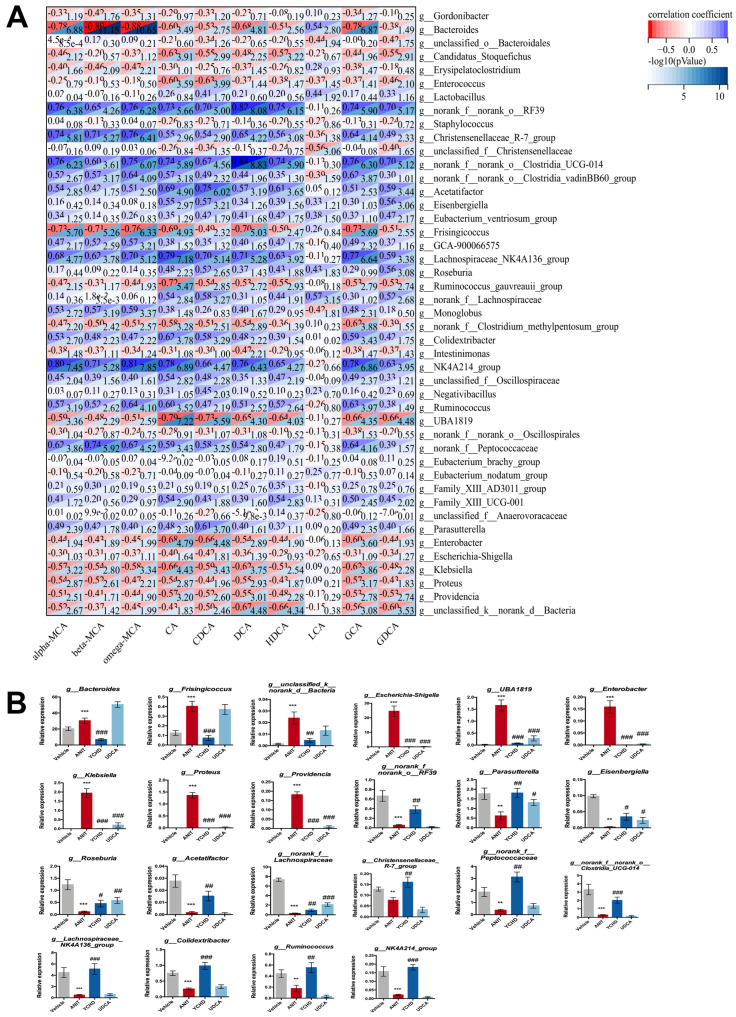
YCHD restores gut microbiota composition in cholestasis. (**A**) BA–microbiota Spearman’s correlation analysis. (**B**) YCHD has regulatory effect on gut microbiota highly correlated with BA metabolism. All values are expressed as mean ± SEM. Compared with Vehicle group, ** *p* < 0.01, *** *p* < 0.001; compared with ANIT group, # *p* < 0.05, ## *p* < 0.01, ### *p* < 0.001.

The gut microbiota plays a key role in converting intestinal BAs to their unconjugated forms and transforming primary BAs into secondary BAs [29]. To investigate the potential regulatory relationship between differential microbial genera and BA metabolism, we performed the Spearman correlation analysis on gut microbiota and BA profiles. Figure 5A demonstrates a significant correlation network between differential genera (LDA > 2, *p* < 0.05) and BAs. The value in the upper-left corner of each grid represents the absolute Spearman correlation coefficient (|ρ|), which suggests a strong correlation when approaching 1. Notably, the non-conjugated BAs—such as CDCA, DCA, and CA—known to act as high-affinity ligand agonists for FXR [29,30], showed significant correlations with most differential bacterial genera in this study.

The Spearman correlation analysis identified bacterial genera significantly modulated by YCHD that exhibited strong associations (|ρ| > 0.5) with at least three BAs (Figure 5B). Compared to the Vehicle group, ANIT challenge induced the upregulation of 9 genera, i.e., *Bacteroides* (+1.5-fold), *Frisingicoccus* (+3.2-fold), *unclassified_k__norank_d_Bacteria* (+14.8-fold), and *Escherichia-Shigella* (+437.5-fold), and the downregulation of 13 genera, including *norank_f__norank_o_RF39* (−12.5-fold), *Parasutterella* (−2.9-fold), *Eisenbergiella* (−46.7-fold), *Roseburia* (−10.6-fold), *Acetatifactor* (−1.7-fold), and *norank_f__Lachnospiraceae* (−23.6-fold). YCHD treatment effectively normalized all 22 dysregulated genera, restoring their abundances to levels comparable to the Vehicle group.

### 2.5. YCHD Improved Intestinal Metabolite Disorders in Mice with Cholestasis

**Figure 6:**
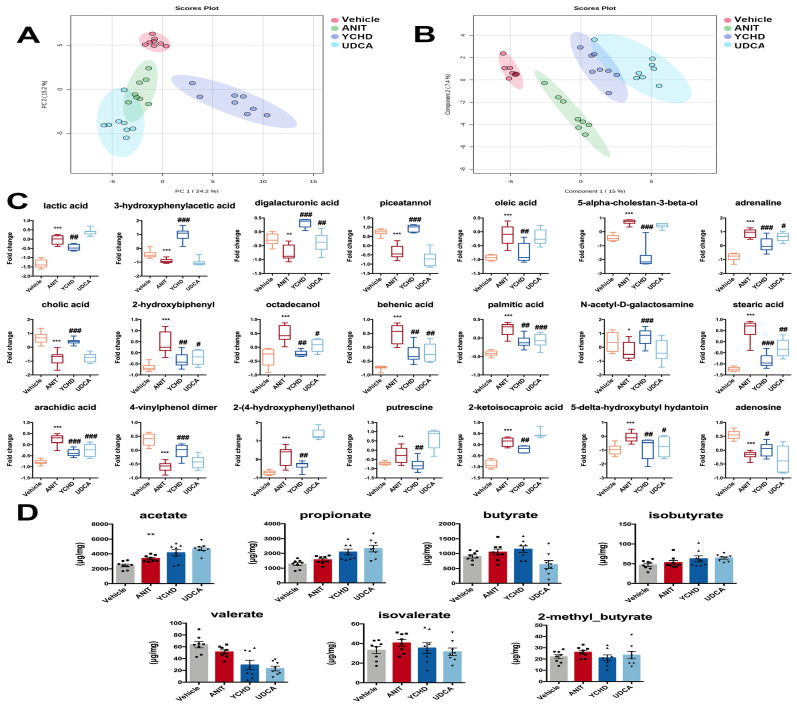
YCHD corrects intestinal metabolic dysregulation. (**A**) Metabolic PCA. (**B**) PLS-DA score plot of intestinal metabolites. (**C**) Intestinal metabolites significantly regulated by YCHD. (**D**) SCFA levels in each group. All values are expressed as mean ± SEM. Compared with Vehicle group, * *p* < 0.05, ** *p* < 0.01, *** *p* < 0.001; compared with ANIT group, # *p* < 0.05, ## *p* < 0.01, ### *p* < 0.001.

Given the microbiota–metabolite axis in cholestasis pathogenesis, we performed complementary metabolomics analyses: non-targeted GC-MS for global metabolite profiling and targeted LC-MS for SCFA quantification. Multivariate analysis (principal component analysis (PCA)/partial least square-discriminant analysis (PLS-DA)) demonstrated distinct metabolic clustering among experimental groups (Figure 6A,B), with YCHD-treated mice exhibiting unique metabolite signatures compared to ANIT-induced cholestasis models.

**Figure 7:**
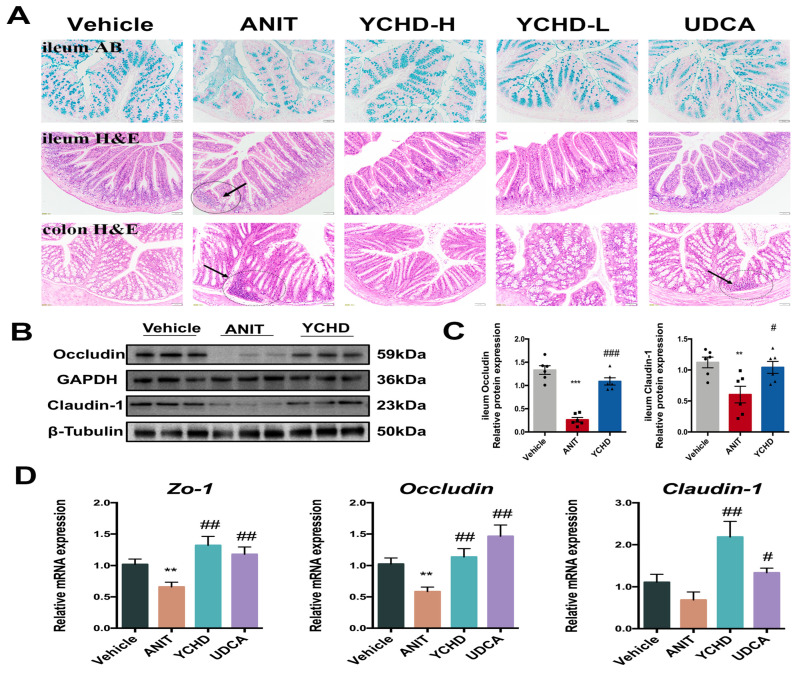
YCHD repairs intestinal barrier integrity. (**A**) Alcian blue staining of ileum samples and H&E staining of ileum and colon samples. Black arrow indicates infiltrated area of ileum and colon. Scale bar: 50 μm. (**B**,**C**) Western blot of Occludin and Claudin-1 in ileum. (**D**) Ileum mRNA expression levels of Zo-1, Occludin, and Claudin-1. All values are expressed as mean ± SEM. Compared with Vehicle group, ** *p* < 0.01, *** *p* < 0.001; compared with ANIT group, # *p* < 0.05, ## *p* < 0.01, ### *p* < 0.001.

Twenty-one differentially regulated metabolites were identified in the YCHD group, including six fatty acids and derivatives such as oleic acid, palmitic acid, stearic acid, and arachidic acid; BA and cholesterol derivatives like cholic acid and 5-alpha-cholestan-3-beta-ol; energy-related metabolites including lactic acid, 2-ketoisocaproic acid, and digalacturonic acid; phenolic metabolites such as piceatannol and 3-hydroxyphenylacetic acid; and sugar and amino acid derivatives exemplified by N-Acetyl-D-galactosamine (Figure 7C). Targeted SCFA analysis revealed no significant alterations across groups (Figure 7D). This indicates that YCHD’s anti-cholestatic effects are likely independent of microbial fermentative metabolism. Crucially, the stability of SCFAs—despite profound microbiota remodeling—reinforces that YCHD primarily targets BA signaling via the FXR-FGF15 axis rather than the general microbial metabolic output.

### 2.6. YCHD Restored Intestinal Mucosal Barrier Disruption in Mice with Cholestasis

Advanced cholestasis induces intestinal mucosal barrier dysfunction, facilitating endotoxin translocation through portal circulation. Histopathological evaluation demonstrated YCHD’s protective effects: (1) Alcian blue staining revealed attenuated ANIT-induced acidic mucin depletion in ileal epithelium, and (2) YCHD significantly reduced inflammatory infiltration in both the ileum and colon (Figure 7A).

The integrity of intestinal tight junction (TJ) critically governs microbial translocation through enterohepatic circulation. Our investigation focused on ileal TJ proteins across three cohorts: Vehicle control, ANIT-induced cholestasis, and high-dose YCHD treatment. ANIT challenge significantly reduced Occludin and Claudin-1 protein expression compared to controls (Figure 7B,C). YCHD intervention effectively reversed this suppression, restoring both proteins to near-physiological levels. Consistent with protein expression patterns, mRNA quantification revealed the coordinated upregulation of *Zo-1*, *Occludin*, and *Claudin-1* in YCHD-treated mice versus ANIT models (Figure 7D). These molecular alterations demonstrate YCHD’s capacity to preserve the intestinal barrier function during cholestatic progression through the transcriptional regulation of TJ components.

### 2.7. Antibiotic Therapy Reduced Efficacy of YCHD in Cholestatic Mice

**Figure 8:**
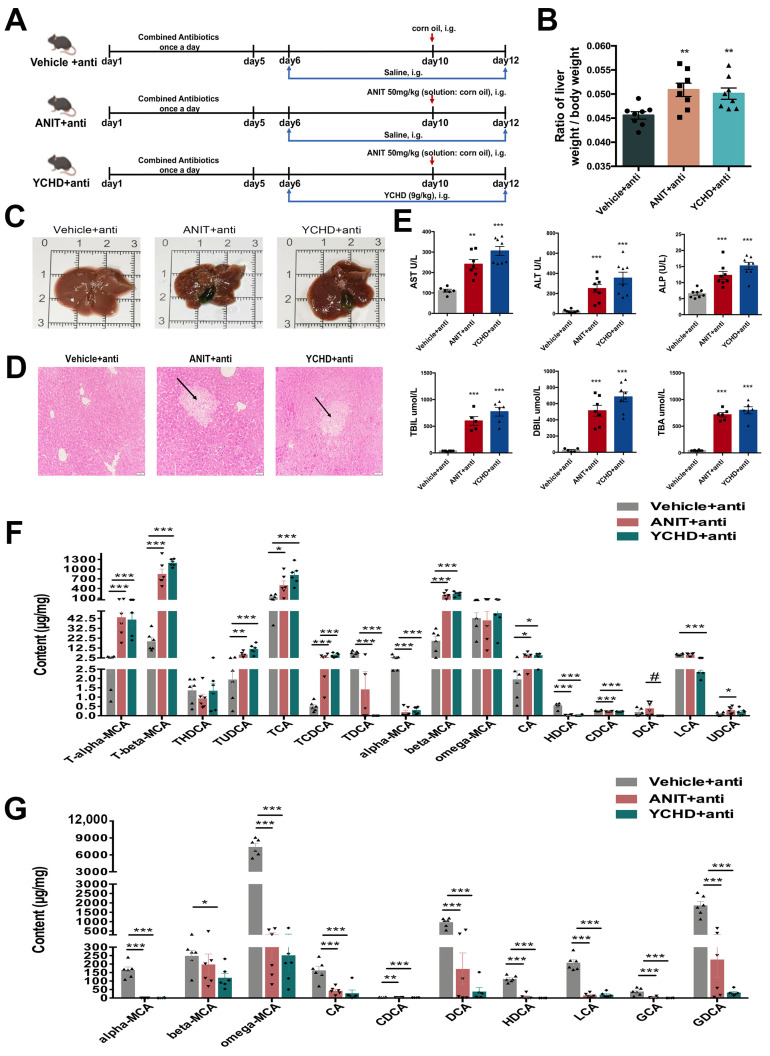
Antibiotic coadministration abolishes YCHD efficacy. (**A**) Experimental design schematic. (**B**) Liver-to-body weight ratios. (**C**) Representative hepatobiliary morphology. (**D**) H&E staining for each group. Scale bar: 50 μm. Black arrow indicates inflammatory infiltration of hepatocytes. (**E**) Serum biomarkers (AST, ALT, ALP, TBIL, DBIL, and TBA). (**F**,**G**) Hepatic/fecal BA profiles. All values are expressed as mean ± SEM. Compared with Vehicle + anti group, * *p* < 0.05, ** *p* < 0.01, *** *p* < 0.001, # *p* < 0.05; compared with ANIT group.

To investigate gut microbiota’s role in YCHD-mediated cholestasis regulation, antibiotic-treated mice with ANIT-induced cholestasis received YCHD coadministration (Figure 8A). The combined treatment showed no therapeutic effects on hepatobiliary parameters: liver-to-body weight ratio remained unchanged (Figure 8B), with persistent gallbladder enlargement and cholestatic symptoms (Figure 8C). Histopathological analysis revealed unaltered hepatocyte injury and inflammatory infiltration (Figure 8D). Serum biomarkers (ALT, AST, ALP, TBA, TBIL, DBIL) showed no improvement (Figure 8E), and YCHD lost its regulatory effects on hepatic and fecal BA metabolism (Figure 8F,G).

**Figure 9:**
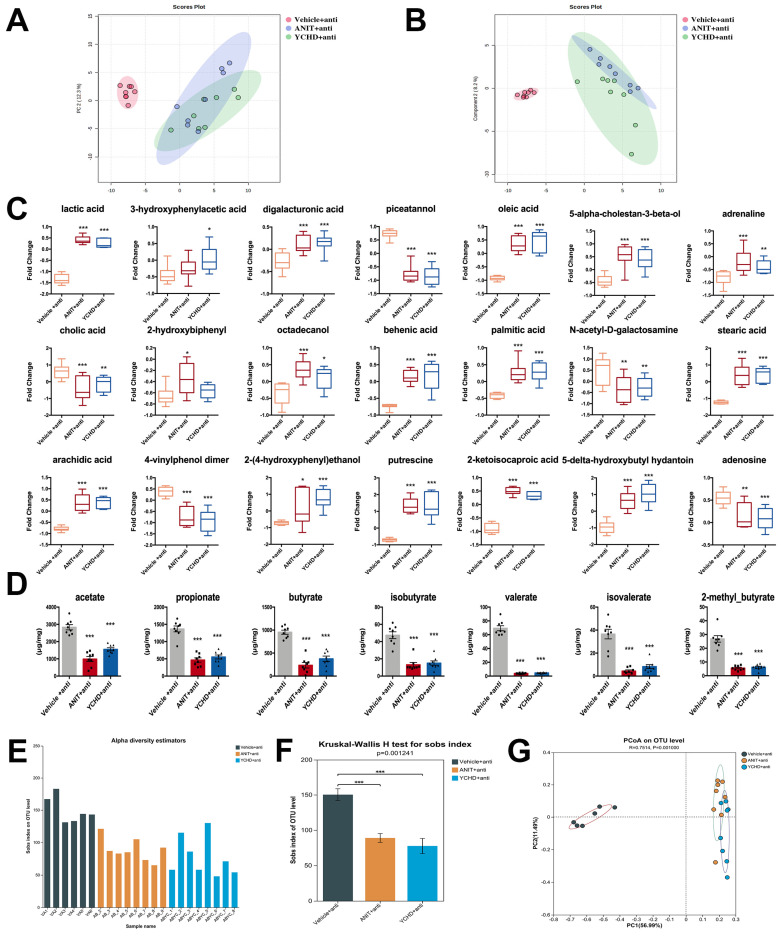
Antibiotics block YCHD-mediated metabolic/microbial regulation. (**A**) Intestinal metabolic PCA. (**B**) PLS-DA of intestinal metabolites. (**C**) Regulatory function of YCHD on intestinal metabolites is lost. (**D**) SCFA levels in each group. (**E**,**F**) α-Diversity indices (Sobs). (**G**) β-Diversity (PCoA). All values are expressed as mean ± SEM. Compared with Vehicle + anti group, * *p* < 0.05, ** *p* < 0.01, *** *p* < 0.001; compared with ANIT group.

Metabolomic profiling demonstrated that YCHD–antibiotic coadministration failed to correct intestinal metabolic disturbances (Figure 9A–C) or restore SCFA homeostasis (Figure 9D). Antibiotic administration significantly reduced bacterial richness, as evidenced by a 72–78% decrease in observed OTUs (Sobs index): ANIT + anti: 95 ± 31 (*p* < 0.001 vs. Vehicle); YCHD + anti: 77 ± 28 (*p* < 0.001 vs. Vehicle). This reduction confirms effective gut microbiota depletion. 16S rRNA sequencing analysis indicated no significant changes in microbial α-diversity indices (Figure 9E,F) or β-diversity patterns (Figure 9G).

### 2.8. FMT from YCHD Alleviated Cholestatic Liver Damage

**Figure 10:**
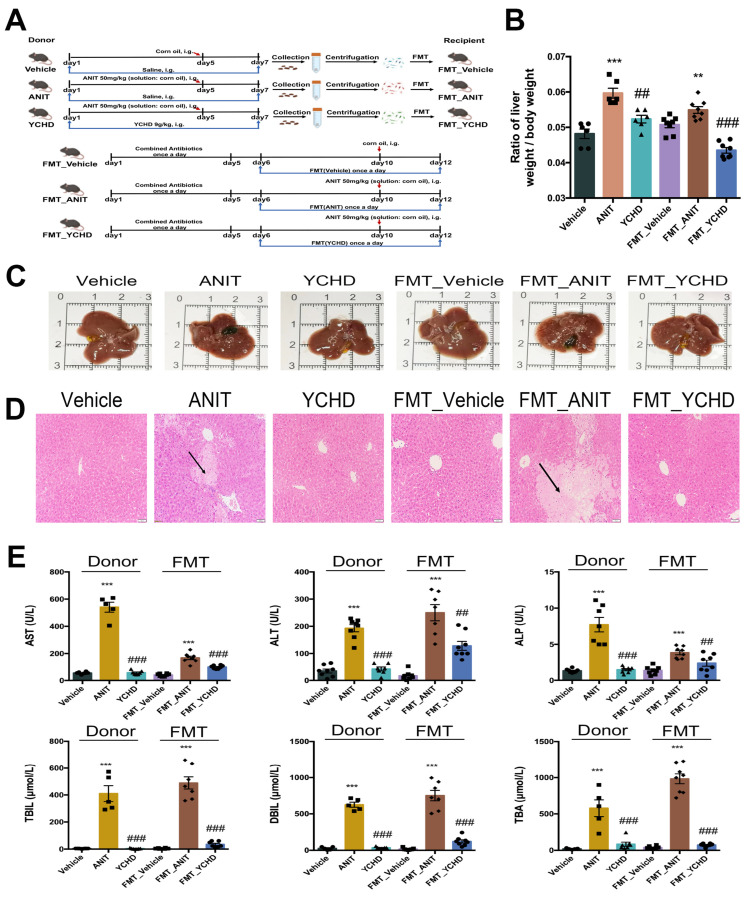
FMT replicates YCHD-mediated hepatoprotection. (**A**) Donor–recipient design. (**B**) Liver-to-body weight ratios. (**C**) Representative hepatobiliary morphology. (**D**) H&E staining for each group. Scale bar: 50 μm. Black arrow indicates inflammatory infiltration of hepatocytes. (**E**) Serum biomarkers (AST, ALT, ALP, TBIL, DBIL, and TBA). All values are expressed as mean ± SEM. Compared with Vehicle + anti group, ** *p* < 0.01, *** *p* < 0.001; compared with ANIT group, ## *p* < 0.01, ### *p* < 0.001.

The FMT experimental design is schematically presented in Figure 10A. Compared to the FMT_Vehicle group, FMT_YCHD administration significantly reduced the elevated liver-to-body weight ratio in FMT_ANIT mice (Figure 10B). Postmortem hepatobiliary analysis demonstrated FMT-mediated improvements in hepatic necrosis and gallbladder distension (Figure 10C), corroborated histologically by reduced hepatocyte necrosis and inflammatory infiltration in H&E-stained sections (Figure 10D). Serum biomarker quantification revealed significant reductions in ALT, AST, ALP, TBIL, DBIL, and TBA levels following FMT intervention (Figure 10E).

### 2.9. FMT Improved BA Disorder in Mice with Cholestasis

**Figure 11:**
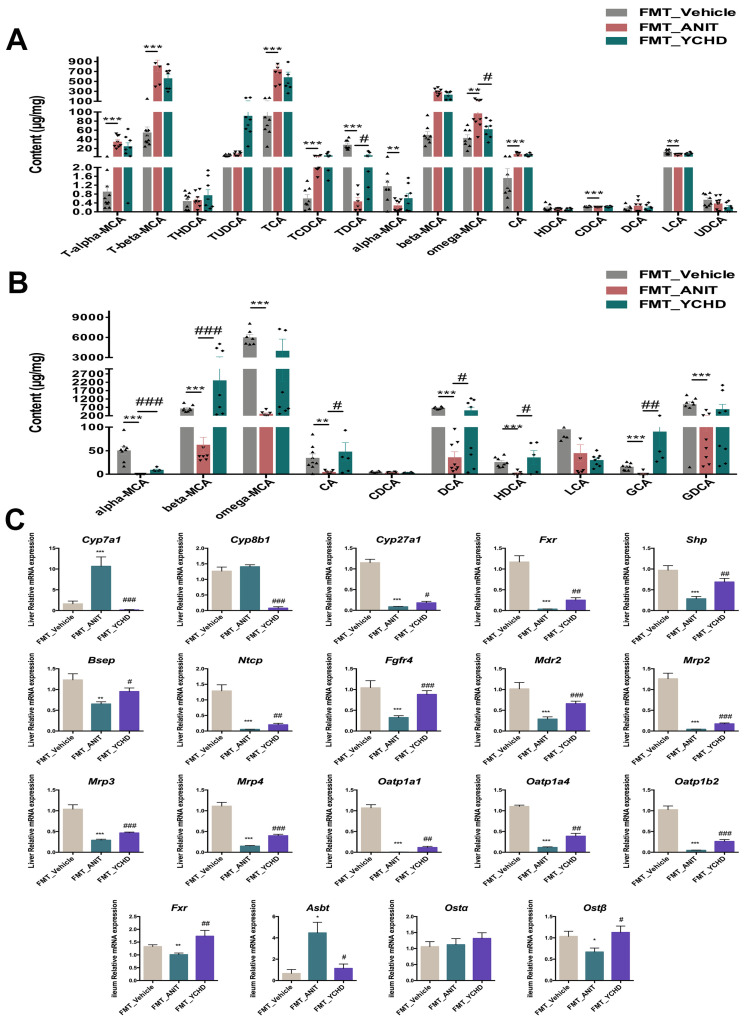
FMT restores BA homeostasis. (**A**) Hepatic BA profiles. (**B**) Fecal BA profiles. (**C**) Hepatic mRNA expression levels of Cyp7a1, Cyp8b1, Cyp27a1, Fxr, Shp, Bsep, Ntcp, Fgfr4, Mdr2, Mrp2, Mrp3, Mrp4, Oatp1a1, Oatp1a4, and Oatp1b2 and ileum mRNA expression levels of Fxr, Asbt, Osta, and Ostβ. All values are expressed as mean ± SEM. Compared with FMT_Vehicle group, * *p* < 0.05, ** *p* < 0.01, *** *p* < 0.001; compared with ANIT group, # *p* < 0.05, ## *p* < 0.01, ### *p* < 0.001.

BA profiles in hepatic and fecal samples from the FMT experiments were quantified using targeted LC-MS. The findings revealed that the majority of BAs, including T-α-MCA, T-β-MCA, TCA, TCDCA, ω-MCA, CA, and CDCA, were markedly elevated in the liver of the FMT_ANIT group. Conversely, a handful of BAs, such as TDCA, α-MCA, and LCA, exhibited a decline. FMT intervention demonstrated limited hepatic BA modulation, achieving only TDCA restoration and ω-MCA reduction (Figure 11A).

Fecal metabolomics identified 10 detectable BAs, with eight species (α-MCA, β-MCA, ω-MCA, CA, DCA, HDCA, GCA, GDCA) exhibiting decreases in the FMT_ANIT group, indicating impaired enterohepatic circulation. FMT treatment reversed this pattern, inducing increases in six critical BAs (α-MCA, β-MCA, CA, DCA, HDCA, GCA), confirming intestinal-targeted therapeutic effects (Figure 11B).

The PCR analysis of BA synthesis/transport enzymes revealed FMT-mediated transcriptional regulation comparable to YCHD oral treatment (Figure 11C). Both interventions significantly suppressed *Cyp7a1* expression (classical synthesis pathway) while upregulating alternative pathway regulator *Cyp27a1* and efflux transporter *Bsep*. The coordinated induction of nuclear receptors *Fxr* and *Shp* established mechanistic consistency between FMT and pharmacological interventions.

### 2.10. FMT from YCHD Significantly Restored Intestinal Homeostasis in Cholestatic Mice

**Figure 12:**
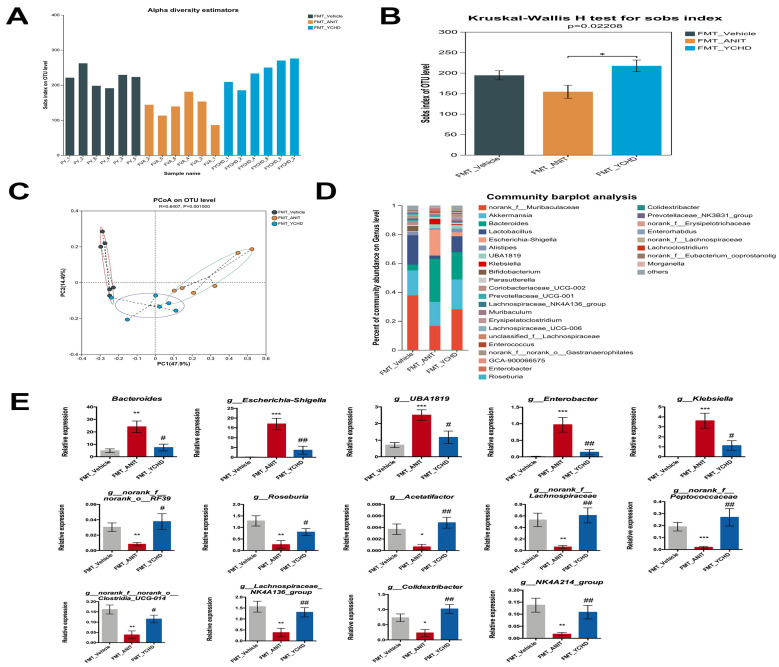
FMT ameliorates intestinal homeostasis disruption. (**A**,**B**) α-Diversity indices (Sobs). (**C**) β-Diversity (PCoA). (**D**) Gut microbiota composition at genus level. (**E**) FMT has regulatory effect on gut microbiota highly correlated with BA metabolism. All values are expressed as mean ± SEM. Compared with FMT_Vehicle group, * *p* < 0.05, ** *p* < 0.01, *** *p* < 0.001; compared with ANIT group, # *p* < 0.05, ## *p* < 0.01.

Given the interplay between intestinal homeostasis and BA metabolism, we investigated FMT’s tripartite regulatory effects on gut microbiota composition, intestinal barrier integrity, and metabolic profiles. 16S rRNA sequencing analysis demonstrated FMT-enhanced microbial α-diversity in ANIT-challenged mice (Figure 12A,B). PCoA revealed distinct clustering patterns among groups (R = 0.64, *p* = 0.001), confirming FMT-induced β-diversity restructuring (Figure 12C). Taxonomic profiling at the genus level showed that FMT_YCHD administration reversed ANIT-induced dysbiosis (Figure 12D). Specifically, in Section 2.4, we identified 22 bacterial genera co-modulated by YCHD that exhibited high relevance to BA metabolism. We further investigated whether these genera were similarly impacted in the FMT experiments. As demonstrated in Figure 12E, 14 of these genera displayed comparable modulatory effects in the FMT group, consistently mirroring the regulatory trends observed with YCHD treatment. For instance, relative to the FMT_ANIT group, FMT from YCHD-treated donors significantly reduced the abundance of detrimental genera, such as *Escherichia-Shigella* (−4.6-fold) and *Bacteroides* (−3.2-fold), while increasing beneficial *Roseburia* (+3.1-fold).

**Figure 13:**
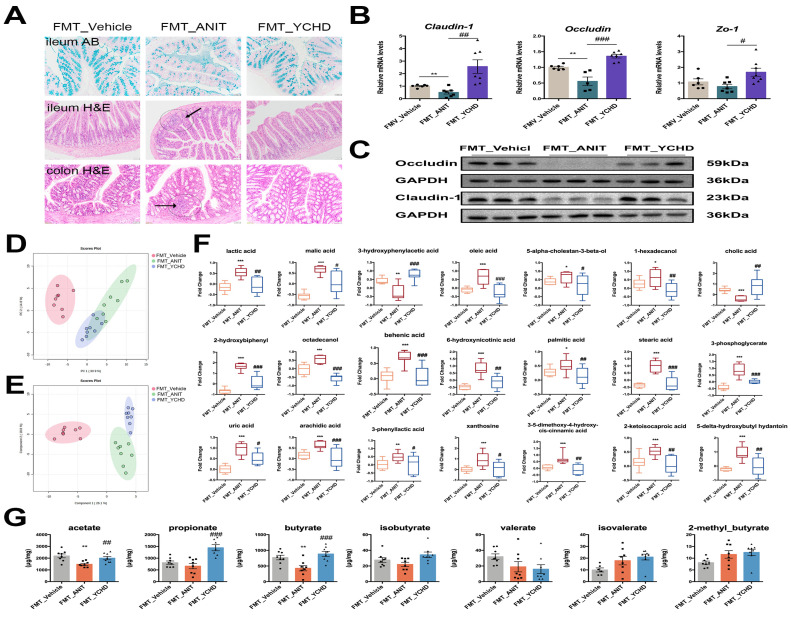
FMT ameliorates intestinal homeostasis disruption. (**A**) Alcian blue staining of ileum samples and H&E staining of ileum and colon samples. Black arrow indicates the infiltrated area of ileum and colon. Scale bar: 50 μm. (**B**) Ileum mRNA expression levels of Zo-1, Occludin, and Claudin-1. (**C**) Western blot of Occludin and Claudin-1 in ileum. (**D**,**E**) PCA and PLS-DA of intestinal metabolites in each group. (**F**) Intestinal metabolites significantly regulated by FMT. (**G**) SCFA levels in each group. All values are expressed as mean ± SEM. Compared with FMT_Vehicle group, * *p* < 0.05, ** *p* < 0.01, *** *p* < 0.001; compared with ANIT group, # *p* < 0.05, ## *p* < 0.01, ### *p* < 0.001.

Alcian blue and H&E staining indicated that FMT therapy effectively countered the reduction in acidic mucin levels in the ileum, as well as the inflammatory cell infiltration in both the ileum and colon, which were induced by ANIT (Figure 13A). Additionally, Western blot and PCR analyses revealed that FMT treatment restored the protein and mRNA expression levels of Occludin and Claudin-1 in the ileum of mice, which had been compromised by ANIT exposure (Figure 13B,C).

To explore FMT’s impact on intestinal metabolite regulation, we conducted untargeted metabolomics on fecal samples. Multivariate analysis (PCA/PLS-DA) showed clear metabolic separation (Figure 13D,E). The specific differential metabolites involved in each group were illustrated in the bar charts. FMT had a modulatory effect on the disordered intestinal metabolites in most of the FMT_ANIT groups (Figure 13F). Notably, FMT restored colonic SCFA production. It could significantly improve the downregulation of intestinal acetate, propionate, and butyrate in the FMT_ANIT group (Figure 13G).

## 3. Discussion

This study establishes a novel mechanism by which YCHD alleviates ANIT-induced intrahepatic cholestasis: the gut microbiota-dependent activation of the FXR-FGF15 pathway. Critically, we demonstrate for the first time that the therapeutic efficacy of this classical herbal formula—the restoration of hepatic function, promotion of BA efflux, suppression of hepatic BA synthesis (Cyp7a1), mitigation of inflammation, and repair of intestinal barrier integrity—is fundamentally dependent on its remodeling of gut microbial communities. Using a multi-layered mechanistic approach integrating 16S rRNA sequencing, targeted BA metabolomics (LC/MS), FMT, and antibiotic depletion, we provide conclusive evidence that YCHD enriches microbiota capable of generating FXR-activating secondary BAs (CDCA, DCA, CA). This microbial restructuring initiates a self-reinforcing cycle: microbiota-derived ligands activate intestinal FXR, triggering FGF15 signaling to suppress hepatic BA synthesis and enhancing hepatobiliary transport, while simultaneously restoring barrier function to stabilize the commensal niche. The abolition of YCHD’s effects by antibiotic depletion and the faithful replication of its full therapeutic profile—including pathway activation, BA homeostasis, and barrier repair—via FMT from YCHD-treated donors unequivocally confirm gut microbiota as the primary mediator driving FXR activation. This work pioneers the causal linkage between a traditional herbal formulation, microbiome restructuring, and nuclear receptor signaling in cholestasis resolution.

While FXR activation represents a key mechanism, YCHD’s anti-cholestatic effects involve a multifaceted network extending beyond nuclear receptor signaling. Our findings demonstrate that YCHD administration not only upregulated hepatic and intestinal FXR/FGF15 expression—shifting BA synthesis from the classical Cyp7a1-dependent pathway toward the alternative Cyp27a1/Cyp8b1 route—but also directly modulated hepatobiliary transport systems independent of FXR signaling. Crucially, we observed the coordinated induction of canalicular efflux transporters (Bsep, Mdr2, Mrp2) and sinusoidal exporters (Mrp3, Mrp4), establishing a hepatoprotective framework that accelerates biliary elimination and systemic redistribution of cytotoxic bile species independent of the FXR-FGF15 crosstalk. This transporter-centric action complements the FXR-mediated suppression of BA synthesis. The incomplete normalization of rodent-specific ω-MCA and α-MCA levels (minor components in human BA pools [31]) likely reflects YCHD’s preferential targeting of the classical Cyp7a1 pathway. While FXR activation by YCHD-derived compounds (e.g., scoparone, geniposide) contributes significantly [32,33,34,35], our integrated analysis revealed microbiota-dependent mechanisms as equally pivotal. Targeted metabolomics showed YCHD-enriched intestinal unconjugated BAs (CDCA, DCA, CA)—potent FXR ligands [29,30]—through the specific restructuring of the gut microbiome.

Gut anaerobic bacteria drive BA metabolism through bile salt hydrolase (BSH)-mediated deconjugation and the bai operon-dependent 7α-dehydroxylation pathway, converting primary BAs to secondary BAs [36]. While our 16S rRNA sequencing approach has inherent limitations in directly resolving functional gene abundance (e.g., bsh and bai genes). Nevertheless, fecal BA profiling provided strong functional evidence: YCHD significantly increased levels of unconjugated BAs (CA, CDCA, α/β/ω-MCA; indicative of enhanced BSH activity) and secondary BAs (DCA, LCA; indicative of enhanced 7α-dehydroxylation). This suggests YCHD remodels a microbiota with enhanced BA-transforming capacity. Critically, these microbiota-derived secondary BAs (e.g., DCA, LCA) serve as potent FXR ligands, activating intestinal FXR-FGF15 signaling to suppress hepatic Cyp7a1. Concurrently, FXR activation induces BA transporters (Bsep, Mrp2) and stabilizes the commensal niche by enriching beneficial taxa while suppressing pathobionts (*Escherichia-Shigella*). The fundamental dependency of this therapeutic axis on the microbiota was unequivocally confirmed by antibiotic ablation abolishing YCHD’s effects (Figure 8 and Figure 9), and FMT from YCHD-treated donors replicating the full therapeutic profile, including BA transformation and FXR activation (Figure 10, Figure 11, Figure 12 and Figure 13). Future studies employing shotgun metagenomics will directly quantify BA metabolism genes (bsh and bai operon) to precisely delineate how YCHD modulates these functional pathways at the genetic level.

YCHD’s modulation of BA-metabolizing bacteria directly underpins its efficacy. Correlation analysis identified 22 genera strongly associated with FXR-activating BAs (Figure 5B). Notably, YCHD selectively suppressed pathogenic taxa including *Escherichia-Shigella* (linked to FXR-SHP pathway inhibition [37]), *Bacteroides* (abnormally enriched in patients with PSC [38]), and pro-inflammatory *Frisingicoccus* (ANIT-induced but functionally uncharacterized); enriched commensal genera such as *Parasutterella* and *Colidextribacter* (critical for BA detoxification and gut barrier integrity [39]), *Eisenbergiella* (essential for FXR-mediated BA enterohepatic circulation [40]), and butyrate-producing taxa like *Roseburia* and *Lachnospiraceae_NK4A136_group* (alleviating cholestasis via butyrate-FXR-FGF15 axis [41]); and restored BA-transforming specialists including *Christensenellaceae_R-7_group* (SCFA producer [42]).

Critically, YCHD-induced microbial remodeling establishes a self-reinforcing virtuous cycle. During the initial phase, the YCHD-mediated modulation of functional bacteria (e.g., *Roseburia*, *Eisenbergiella*) generates secondary BAs (e.g., CDCA/DCA) that activate intestinal FXR. This FXR activation triggers FGF15 secretion, subsequently suppressing hepatic Cyp7a1 expression. In the subsequent phase, FXR pathway activation reciprocally sustains commensal ecology—stabilizing the colonization of barrier-enhancing taxa like *Parasutterella* and *Colidextribacter*, while eliminating pathogenic bacteria (e.g., *Escherichia-Shigella*) that inhibit the FXR pathway and disrupt intestinal TJ proteins. Future studies employing gnotobiotic animal models (e.g., co-colonization with *Roseburia* and *Eisenbergiella*) could precisely dissect the causal contributions of specific bacterial species to BA metabolism, advancing targeted microbiome-based precision therapeutics.

Cholestatic liver disease induces gut microbiota dysbiosis and impairs intestinal barrier function, with elevated intestinal permeability exacerbating inflammatory responses and disease progression [43,44]. In ANIT-induced cholestatic mice, intestinal inflammation and mucus barrier disruption were attenuated by YCHD and FMT. TJ proteins—including Zo-1, Occludin, and Claudin-1—maintain intestinal epithelial integrity, whose dysfunction compromises mucosal immunity [45]. YCHD restored the ANIT-induced downregulation of these TJ proteins, indicating intestinal barrier preservation. Given that impaired FXR signaling exacerbates barrier dysfunction in chronic liver diseases and FXR agonists enhance TJ protein expression [46], YCHD-mediated FXR activation likely contributes to its barrier-protective effects.

Beyond gut microbiota, microbial metabolites have gained increasing research attention. However, the functional roles of most gut-derived metabolites remain poorly characterized, with their identities and biological activities largely undefined [47]. These metabolites are broadly categorized into three groups: (1) dietary components metabolized by gut bacteria (e.g., SCFAs); (2) host-derived molecules modified by microbiota (e.g., BAs); and (3) de novo bacterial products (e.g., branched-chain amino acids, polyamines, vitamins) [48]. Untargeted metabolomics revealed YCHD’s global correction of ANIT-induced metabolic dysregulation. However, the functional annotation of specific metabolites was limited by the current knowledge gaps. Our findings demonstrate that YCHD upregulates intestinal cholic acid levels, which directly activates intestinal FXR to suppress hepatic Cyp7a1 expression and reduce BA synthesis—consistent with the observed FXR-FGF15 pathway activation. Concurrently, the increased abundance of *N*-acetyl-D-galactosamine, a core component of mucin O-glycans, suggests enhanced mucus layer synthesis and strengthened physical barrier function [49], aligning with the upregulated TJ protein expression. The phenolic compound 3-hydroxyphenylacetic acid—a microbial metabolite of dietary polyphenols (e.g., flavonoids) [50]—exerts anti-inflammatory and antioxidant activities, potentially inhibiting NF-κB signaling pathways, which corroborates our prior mechanistic studies [51]. Reduced putrescine levels indicate the suppression of pathogenic bacterial overgrowth (e.g., *Escherichia-Shigella*), given its barrier-disruptive effects at high concentrations [52]. Collectively, these metabolic shifts demonstrate that YCHD restores intestinal homeostasis in cholestatic mice by remodeling microbiota functionality to generate protective metabolites. Notably, YCHD did not alter SCFA levels despite reshaping microbiota composition (Figure 6D). This aligns with its phytochemical-rich nature that lacks fermentable fibers, and reinforces that its therapeutic effects operate primarily through BA signaling (FXR activation, BA transporter regulation) rather than global microbial metabolism.

It should be noted that the experimental model established using ANIT in this study may exert multifaceted impacts on host metabolic pathways. While our experimental design prioritized clinical relevance by mimicking prophylactic/therapeutic YCHD administration, potential interactions between YCHD components and ANIT metabolism warrant further exploration. Phytochemicals in YCHD (e.g., geniposide [53], scoparone [54]) may influence ANIT bioactivation or clearance through the modulation of hepatic detoxification enzymes (e.g., CYP450 isoforms [55]), thereby indirectly affecting cholestatic outcomes. Although microbiota-depletion and FMT experiments support the gut-dependent mechanism of YCHD, direct herb–toxin interactions cannot be excluded. Future studies should integrate pharmacokinetic analyses—such as the LC-MS/MS quantification of YCHD phytochemicals and ANIT metabolites in serum and tissues—to assess co-exposure dynamics. In vitro hepatocyte models or liver microsomal assays could further dissect YCHD’s impact on ANIT metabolic pathways, while humanized mouse models may clarify interspecies differences in detoxification. Multi-omics approaches may also reveal YCHD-induced shifts in detoxification networks (e.g., Nrf2, GSTs) that modulate ANIT toxicity. Collectively, these investigations will delineate whether YCHD’s efficacy stems solely from the microbiota–FXR crosstalk or synergizes with direct xenobiotic regulation, ultimately optimizing its clinical application.

The findings of this study suggest that YCHD holds significant promise as alternative therapy for cholestatic liver diseases. Current first-line treatments, such as UDCA and OCA, fail to achieve adequate biochemical or histological responses in approximately 50% of patients [2,3]. YCHD’s unique mechanism—modulating gut microbiota and activating the FXR-FGF15 pathway—addresses key pathological features of cholestasis, including BA dysregulation, gut barrier dysfunction, and microbial dysbiosis. This multi-target action positions YCHD as a potential adjunct to existing therapies. For instance, combining YCHD with UDCA or OCA could synergistically enhance BA efflux, reduce hepatotoxicity, and improve treatment resistance, particularly in patients with incomplete responses to monotherapy. Clinically, YCHD may benefit specific populations, such as pregnant women with intrahepatic cholestasis of pregnancy (ICP), where conventional therapies are limited due to safety concerns. A previous clinical meta-analysis of 3841 patients supports YCHD’s efficacy in reducing pruritus and serum BA levels in ICP patients [19]. Additionally, YCHD’s natural composition and historical use in traditional medicine may offer a favorable safety profile for the long-term management of chronic cholestatic conditions like PBC or PSC. However, translating these preclinical findings into clinical practice requires addressing several challenges. First, the complexity of YCHD’s herbal components necessitates the standardization of active compounds (e.g., geniposide, scoparone) to ensure consistency and reproducibility. Second, clinical trials are urgently needed to validate YCHD’s efficacy, optimal dosing, and safety in humans. Third, the interplay between YCHD and gut microbiota underscores the importance of personalized approaches, as individual microbial profiles may influence therapeutic outcomes. Future studies should explore the microbiome-guided stratification of patients to maximize treatment responsiveness. To advance the clinical translation of YCHD, three prioritized research directions should be pursued: first, establishing dual quality control criteria that simultaneously monitor core phytochemical constituents (geniposide/scoparone) and functional microbiota abundance (e.g., Roseburia), ensuring batch-to-batch consistency in microbiotal modulatory activity; second, conducting gut microbiome-stratified clinical trials evaluating YCHD as an add-on therapy for cholestatic patients refractory to UDCA; and third, developing engineered bacterial therapeutics (e.g., *Escherichia coli* Nissle 1917 expressing BA hydrolase genes) for the targeted delivery of secondary BAs and butyrate, circumventing the phytochemical complexity inherent to multi-herbal formulations.

Our study also has limitations that require consideration. First, while FMT from YCHD-treated mice replicated therapeutic effects, residual phytochemicals in donor fecal material could confound these observations. Although antibiotic depletion experiments confirmed the necessity of gut microbiota for YCHD’s efficacy, antibiotics may exert off-target effects on hepatic metabolism and immune responses [56], complicating mechanistic interpretations. To address this limitations, future studies will utilize gnotobiotic models combined with defined microbial consortia colonization. This approach will eliminate interference from residual compounds and antibiotic-related artifacts, enabling the precise dissection of microbiota-specific contributions to YCHD’s efficacy. Second, the exclusive use of male mice avoids confounding from estrogen fluctuations but limits extrapolation to female populations, particularly for estrogen-sensitive conditions like ICP. This study was limited to mouse model, and the results may not fully apply to humans, and clinical trials are urgently needed to validate YCHD’s efficacy, optimal dosing, and safety in humans. Finally, although our findings establish a critical link between YCHD-mediated gut microbiota remodeling and FXR-FGF15 pathway activation, several mechanistic questions remain unresolved. While we observed correlations between specific bacterial taxa (e.g., *Roseburia*, *Parasutterella*) and secondary BAs (e.g., DCA, CDCA), the exact microbial enzymes (e.g., BSH, 7α-dehydroxylases) responsible for BA biotransformation were not directly identified. In addition, the causal relationship between microbiota-derived metabolites and FXR signaling requires further validation—whether these metabolites act as direct FXR ligands or modulate receptor activity through intermediate pathways remains unclear. To address these gaps, future studies should employ multi-omics integration, combining metagenomics (to pinpoint bacterial enzymes) with fecal metabolomics (to track metabolite dynamics). Gnotobiotic models colonized with defined microbial consortia could isolate the contributions of specific taxa to BA metabolism and FXR activation. Additionally, in vitro systems (e.g., FXR reporter assays coupled with microbial metabolite screening) may identify novel FXR agonists derived from YCHD–microbiota interactions. While our focus centered on the FXR-FGF15 axis, crosstalk with other nuclear receptors (e.g., PXR, VDR) or inflammatory pathways (e.g., NF-κB) warrants exploration. Such investigations will provide a holistic understanding of YCHD’s multi-target action and accelerate its translation into precision therapies for cholestatic disorders.

## 4. Materials and Methods

### 4.1. Preparation of YCHD

YCHD used in this study was prepared in-house under standardized protocols. YCHD consists of three herbal ingredients derived from nature: *Artemisia capillaris* (*Yinchen*, aerial part), *Gardenia jasminoides* (*Zhizi*, fruit part), and *Rheum palmatum* (*Dahuang*, roots and rhizomes). All the ingredients were provided by Shuguang Hospital, which is affiliated with Shanghai University of Traditional Chinese Medicine. YCHD was prepared by the method recorded in *Shanghan Lun* through the following processes: First, *Artemisia capillaris* (36 g) was boiled in 2.8 L distilled water until the solution volume was reduced to 1.4 L, then *Gardenia jasminoides* (18 g) and *Rheum palmatum* (12 g) were added, kept boiling for 10 min, and the mixture was passed through filter paper. Finally, the filtrate was evaporated under reduced pressure at 60 °C to a concentration of 1.0 g crude drug/mL. YCHD was packaged and stored at −20 °C prior to the studies. To ensure batch homogeneity, all experiments utilized a single GMP-produced batch of YCHD extract (Lot YCHD202401SH).

### 4.2. Identification of Constituents in YCHD by Ultrahigh-Performance Liquid Chromatography–Mass Spectrometry (UHPLC-MS)

YCHD extracts were analyzed on an Agilent 1290 UHPLC system paired with a 6530B Q/TOF mass spectrometer. The setup featured a BEH C18 column (100 × 2.1 mm, 1.7 μm, from Waters, Milford, MA, USA), operating at a temperature of 40 °C within the column compartment. The mass spectra were recorded in positive electrospray ionization (ESI) mode, utilizing an all-ion fragmentation (AIF) approach across a mass range of m/z 100 to 1700. The gas temperature was maintained at 350 °C, with the sheath gas set at 300 °C. The mobile phase consisted of A: H_2_O with 0.1% formic acid, and B: acetonitrile with 0.1% formic acid. Samples were eluted at a flow rate of 0.3 mL/min following a gradient profile: from 5% B to 55% B over the first ten minutes, then ramping from 55% B to 95% B between 10 and 20 min, followed by a 5 min isocratic hold at 95% B, and concluded by a 3 min post-run period. The injection volume was set at 5 μL.

### 4.3. Mice

Male C57BL/6J mice, aged six weeks, were acquired from SLAC Laboratory Animal Inc. in Shanghai, China. Prior to the study, the mice underwent a one-week acclimation period under controlled conditions: temperatures of 23–24 °C, humidity levels between 0–60%, and a 12 h light–dark cycle. They were provided with unrestricted access to water and standard chow. All experimental procedures, including oral gavage interventions, were carried out in compliance with the Animal Experiment Guidelines of Shanghai University of Traditional Chinese Medicine. The study protocol received approval from the university’s Animal Research Ethics Committee (approval number: PZSHUTCM211129013).

Based on the standard clinical dosage of YCHD for a 70 kg adult (66 g crude drug/day), the equivalent mouse dose was calculated using body surface area (BSA) normalization. Specifically, mouse dose (g/kg) = human dose (g/kg) × (human km factor/mouse km factor), where human dose = 66 g/70 kg = 0.943 g/kg, human km factor = 37, and mouse km factor = 3 [57]. Thus, 0.943 g/kg × (37/3) = 11.63 g/kg. The high dose (9 g/kg) represents ~80% of this calculated equivalent to ensure safety [58], while the low dose (3 g/kg) reflects proportional scaling.

In line with our established intrahepatic cholestasis model [59], mice were fasted overnight and administered a single dose of ANIT (50 mg/kg in corn oil) or Vehicle control via gavage. Male C57BL/6J mice received oral YCHD (3 or 9 g/kg) or saline daily for 7 days. Critically, to evaluate both prophylactic and therapeutic potentials, we designed a dual-phase intervention. Prophylactic phase: YCHD pretreatment on Days 1–4 (before ANIT challenge). Therapeutic phase: continued YCHD coadministration on Days 5–7 (during ANIT-induced injury). ANIT was administered on Day 5 to model clinical scenarios where herbal interventions begin before/during toxin exposure (e.g., drug-induced cholestasis). This timing aligns with ANIT-induced injury peak at 48 h (Day 7) [26], coinciding with YCHD’s therapeutic window. Positive control mice received UDCA (50 mg/kg/day) throughout the 7-day regimen. Throughout the 7-day administration period (YCHD 3–9 g/kg/day), no adverse effects—including diarrhea, weight loss, allergic reactions, or mortality—were observed.

### 4.4. Analysis of Serum Biochemical Markers

Upon the completion of the study, blood samples were drawn from the mice to evaluate key serum biomarkers, including aspartate aminotransferase (AST), alanine aminotransferase (ALT), total bile acid (TBA), total bilirubin (TBIL), direct bilirubin (DBIL), and alkaline phosphatase (ALP). The necessary assay kits for these measurements were sourced from Jiancheng Bioengineering Inc., located in Nanjing, China.

### 4.5. Histopathology

Liver, ileum, and colon tissue samples were preserved in 10% neutral buffered formalin, processed into paraffin blocks, and sliced into 5-micron-thick sections. These sections were then stained with hematoxylin and eosin (H&E) to identify necrotic areas and assess any structural abnormalities. Additionally, intestinal tissues were fixed using 4% Carnoy’s Fluid (Adamas Life, Shanghai, China), embedded in paraffin, and similarly sectioned at 5 microns. To detect acid mucin, an Alcian Blue staining kit (Vectorlabs, Beijing, China) was employed on the prepared tissue sections.

### 4.6. Western Blot Analysis

Samples of the liver and ileum were broken down in RIPA buffer (Solarbio, Norton, VA, USA) spiked with 1% PMSF (Beyotime, Shanghai, China). Then, the total protein concentration in these samples was measured. A total of 15 milligrams of protein was mixed into SDS-PAGE sample loading buffer (Beyotime, China). This protein–buffer mixture was then run on 10–15% SDS-PA gels (Beyotime, China) to separate the proteins. After that, the separated proteins were transferred onto pure PVDF membranes (Millipore, Burlington, MA, USA). The HRP-conjugated secondary antibodies were allowed to incubate at room temperature for one hour, after which the signals were visualized using the ECL Chemiluminescent Substrate Kit from Beyotime, China. The resulting images were then processed and analyzed using a multifunctional imaging system manufactured by ProteinSimple in the San Jose, CA, USA.

The information of antibodies were as follows: FXR (25055-1-AP, 1:1000, Proteintech), FGF-15 (sc-398338, 1:100, Santa Cruz), CYP7A1 (18054-1-AP, 1:1000, Proteintech), CYP27A1 (14739-1-AP, 1:1000, Proteintech), Occludin (27260-1-AP, 1:1000, Proteintech), Claudin-1 (13050-1-AP, 1:1000, Proteintech), GAPDH (60004-1-AP, 1:50,000, Proteintech), β-Tubulin (10094-1-AP, 1:4000, Proteintech), HRP-conjugated Goat Anti-Rabbit IgG (H+L) (SA00001-2, 1:5000, Proteintech), and HRP-conjugated Goat Anti-Mouse IgG (H+L) (SA00001-1, 1:5000, Proteintech).

### 4.7. Quantitative Real-Time Polymerase Chain Reaction (RT-PCR)

Total RNA was isolated from liver and ileum tissues using a commercially available tissue RNA extraction kit, following the manufacturer’s protocol (Vazyme, Nanjing, China). RT-PCR analysis was carried out on a QuantStudio 3 Real-Time PCR System (Thermo Fisher Scientific, Waltham, MA, USA) with SYBR Green^®^ Premix DimerEraserTM (Takara, Kusatsu City, Japan) as the detection reagent. To ensure accuracy, the data were normalized to β-actin expression levels and analyzed using the 2^−ΔΔCT^ method. The specific primer sequences utilized in this study are detailed in Table 2.

### 4.8. BAs Assessment

BAs in the liver and fecal content were quantitatively measured based on a modified as described previously [59].

### 4.9. Quantification of Short-Chain Fatty Acid (SCFA) Profiles

In this study, we quantified short-chain fatty acids (SCFAs) such as acetate, propionate, butyrate, isobutyrate, valerate, isovalerate, and 2-methyl-butyrate from fecal samples using a DIONEX LC-MS/MS system based in Sunnyvale, CA, USA. Initially, 50 mg of fecal material was mixed with 0.5 mL of 50% acetonitrile and processed in a homogenizer for 5 min. Following this, the homogenized mixture underwent centrifugation at 13,200 rpm for a duration of 10 min. Subsequently, 40 μL of the resulting supernatant was pipetted into a 1.5 mL tube, where it was combined with 5 μL of an internal standard solution (10 μg/mL d3-caproic acid). Next, the mixture received 20 μL of a 200 mM solution of 3-nitrophenylhydrazine (3NPH), which was prepared by dissolving 37.92 mg of 3NPH in 1 mL of 50% acetonitrile. Additionally, 20 μL of a 120 mM solution of EDC, made by dissolving 23 mg of EDC in 50% acetonitrile with 6% pyridine, was incorporated. This concoction was then incubated at 40 °C for 30 min and subsequently centrifuged again at 13,200 rpm for 10 min. To the supernatant, 180 μL of 50% acetonitrile was added and mixed thoroughly by vortexing. Finally, the mixture was spun at 18,000 rpm for another 10 min, and a 100 μL aliquot of the supernatant was collected for the LC-MS/MS analysis. The SCFA standard mix included acetate, propionate, butyrate, isobutyrate, valerate, isovalerate, 2-methyl-butyrate, and d3-caproic acid, and their concentrations were ascertained through an internal standard method using a standard curve.

### 4.10. GC-MS-Based Metabolomics

Details regarding the fecal sample handling, derivatization of the samples, instrument settings, and data analysis protocols can be found in our earlier work [60].

### 4.11. Fecal Microbiota Transplantation (FMT)

#### 4.11.1. Antibiotic Pretreatment of Recipient Mice

FMT was carried out following protocols established in a prior study [61]. Recipient mice (FMT_Vehicle, FMT_ANIT, FMT_YCHD) received an antibiotic cocktail via daily intragastric gavage (200 μL/day) for 5 consecutive days to deplete endogenous microbiota. The cocktail composition and administration were as follows: vancomycin hydrochloride (Gram-positive coverage): 500 mg/kg/day; metronidazole (Anaerobic coverage): 500 mg/kg/day; neomycin sulfate (Gram-negative coverage): 500 mg/kg/day; and ampicillin sodium salt (extended-spectrum): 500 mg/kg/day. Antibiotics were dissolved in sterile PBS (pH 7.4) and filter-sterilized (0.22 μm). Fresh cocktail was prepared daily and delivered using a 20G ball-tipped gavage needle. FMT was initiated 24 h after the final antibiotic dose.

#### 4.11.2. Donor Fecal Material Processing

Fresh fecal pellets (100 mg) from donor groups (Vehicle, ANIT, YCHD) were immediately suspended in 600 μL ice-cold sterile PBS (0.1 M, pH 7.4; Corning, Corning, NY, USA) within 5 min of defecation. The suspension was homogenized by vortexing (1 min) followed by mechanical disruption (30 s at 30 Hz, TissueLyser II, Qiagen, Hilden, Germany), filtered through a 40 μm nylon cell strainer (Corning, Corning, NY, USA), and centrifuged at 500× *g* for 3 min at 4 °C. The bacteria-enriched supernatant was collected and kept on ice, with all procedures completed within 15 min of fecal collection to preserve microbial viability.

#### 4.11.3. FMT Procedure and Cholestasis Induction

Recipient mice received 100 μL of freshly prepared supernatant via daily intragastric gavage for 7 days, with each aliquot prepared within 10 min prior to administration. On day 5 of FMT, after overnight fasting, the FMT_ANIT and FMT_YCHD groups received a single dose of ANIT (50 mg/kg in corn oil; Sigma-Aldrich, St. Louis, MI, USA) to induce cholestasis, while the FMT_Vehicle control group received volume-matched corn oil.

### 4.12. Antibiotic Cocktail Experiment

To deplete the gut microbiota in mice, a combination of broad-spectrum antibiotics—neomycin sulfate (200 mg/kg), ampicillin (200 mg/kg), metronidazole (200 mg/kg), and vancomycin (100 mg/kg)—was administered orally once daily over a span of five consecutive days. Starting on the 6th day and continuing through the 12th day, the YCHD + anti group received a high-dose oral administration of YCHD (9 g/kg) once daily. Meanwhile, the Vehicle + anti and ANIT + anti groups were given an equivalent volume of saline. On the 10th day, after an overnight fast, the ANIT + anti and YCHD + anti groups were administered a single dose of ANIT (50 mg/kg), dissolved in corn oil, via gavage to induce cholestasis. In contrast, the Vehicle + anti group received the same volume of corn oil without ANIT to serve as the control.

### 4.13. 16S rRNA Gene Sequencing

#### 4.13.1. DNA Extraction and PCR Amplification

Total microbial genomic DNA was extracted from fecal samples using the FastPure Stool DNA Isolation Kit (MJYH, Nanjing, China) following manufacturer’s protocol. DNA quality was verified by 1.0% agarose gel electrophoresis and quantified with NanoDrop^®^ ND-2000 spectrophotometer (Thermo Scientific, Waltham, MA, USA). The V3-V4 hypervariable region of bacterial 16S rRNA gene was amplified using primers 338F (5′-ACTCCTACGGGAGGCAGCAG-3′) and 806R (5′-GGACTACHVGGGTWTCTAAT-3′) on a GeneAmp PCR System 9700 (Applied Biosystems, Waltham, MA, USA). The 20 μL reaction contained the following: 4 μL 5× Fast Pfu buffer, 2 μL 2.5 mM dNTPs, 0.8 μL each primer (5 μM), 0.4 μL Fast Pfu polymerase, and 10 ng template DNA. Cycling conditions: 95 °C/3 min; 27 cycles of 95 °C/30 s, 55 °C/30 s, 72 °C/45 s; and final extension at 72 °C/10 min. Triplicate PCR products were gel-purified and quantified (Synergy HTX, Biotek, Winooski, VT, USA).

#### 4.13.2. Illumina Sequencing

Purified amplicons were pooled equimolarly and paired-end sequenced (2 × 300 bp) on the Illumina MiSeq platform (Illumina, San Diego, CA, USA) by Majorbio Bio-Pharm Technology Co., Ltd. (Shanghai, China). Average sequencing depth was 50,000 reads per sample.

#### 4.13.3. Data Processing and Statistical Analysis

Raw FASTQ files were processed through QIIME2 (v2021.11) pipeline: Quality control: Truncated at 250 bp (Q-score < 20 threshold) and denoised using DADA2 with error-correction modeling; ASV generation: Amplicon Sequence Variants (ASVs) were derived replacing OTU clustering; taxonomic assignment: Silva v138 database via q2-feature-classifier (confidence threshold 0.7); filtering: chloroplast/mitochondrial sequences removed; rarefaction: subsampled to 20,000 sequences/sample (Good’s coverage > 99%).

All analyses performed on the Majorbio Cloud Platform (https://cloud.majorbio.com): alpha diversity: observed ASVs, Chao1, Shannon indices (QIIME2); beta diversity: Bray–Curtis PCoA with PERMANOVA testing (Vegan v2.4.3); differential taxa: LEfSe analysis (LDA > 2, *p* < 0.05); network analysis: co-occurrence networks (Spearman |ρ| > 0.5, *p* < 0.05).

### 4.14. Statistical Analysis

We carried out statistical analysis using Prism 9.0 (GraphPad Software, San Diego, CA, USA). The data were shown as the mean ± standard error of the mean (SEM). To assess the statistical differences between two groups, we employed a two-tailed unpaired Student’s *t*-test. For groups numbering more than two, we used one-way ANOVA for statistical evaluation. A *p*-value of less than 0.05 was regarded as statistically significant. Correlations between different bacteria taxa and BAs were evaluated using Spearman’s correlation analysis with the “heatmap” package in R (version 3.3.1). *p*-value ≤ 0.05 was considered to be statistically significant.

## 5. Conclusions

**Figure 14:**
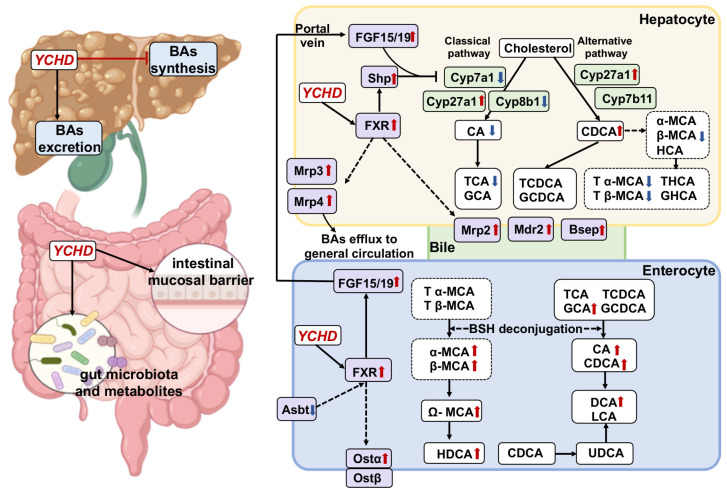
Schematic illustration of mechanism. YCHD alleviates cholestasis through coordinated regulation of hepatic BA transport, suppression of BA synthesis, gut microbiota modulation, and intestinal barrier repair, mediated via FXR-FGF15 enterohepatic axis.

This study employs a multi-dimensional analysis of gut–liver axis regulation to systematically elucidate the therapeutic mechanism by which YCHD ameliorates ANIT-induced cholestatic liver injury. YCHD activates the hepatic–intestinal FXR-FGF15 pathway, markedly suppressing Cyp7a1-mediated BA synthesis while enhancing BA efflux via Bsep/Mrp transporters, thereby restoring BA homeostasis (Figure 14). Crucially, YCHD-remodeled gut microbiota—characterized by the promoted proliferation of beneficial taxa (e.g., *Roseburia*, *Parasutterella*) and the suppressed colonization of pathogenic species (e.g., *Escherichia-Shigella*)—directly drives the generation of secondary BAs (CDCA/DCA). Antibiotic-mediated microbiota ablation abolished YCHD’s efficacy, whereas FMT from YCHD-treated mice recapitulated therapeutic outcomes, establishing gut microbiota as indispensable for YCHD’s action. This “microbiota–BA–FXR” regulatory circuit establishes a novel paradigm for the modern pharmacological investigation of traditional formulations. However, several limitations warrant further investigation: First, despite FMT confirming the microbiota’s critical role, residual phytochemicals in donor feces may confound result interpretation. Though antibiotic validation supports microbiota necessity, the off-target effects of antibiotics on hepatic metabolism require exclusion. Second, the exclusive use of male mice eliminates estrogen interference but restricts extrapolation to estrogen-sensitive conditions like ICP. Third, while specific bacterial genera (e.g., *Roseburia*) correlate with secondary BA metabolism, enzymatic drivers and direct causal links between microbial metabolites and FXR signaling remain unverified. Future research will prioritize the following: employing gnotobiotic models colonized with defined consortia to eliminate phytochemical interference and precisely dissect microbial contributions; extending validation to female animal models and clinical studies evaluating YCHD in sex-specific disorders (e.g., ICP); and integrating metagenomics and metabolomics to identify BA-transforming enzymes and screen novel FXR agonists.

## Figures and Tables

**Table 1:** Results of LC-MS/MS analysis of YCHD.

No.	Chemical Formula	Retention Time (min)	*m*/*z*	Chemical Name	PubChem Compound Identifier
1	C_7_H_12_O_6_	0.858	215.0517	Quinic acid	6508
2	C_16_H_14_O_4_	0.858	309.0494	Ammidin	10212
3	C_16_H_24_O_11_	0.958	431.0919	Caryoptosidic acid	131751540
4	C_17_H_22_O_10_	0.992	425.0876	1-O-Sinapoylglucose	6168296
5	C_16_H_26_O_9_	0.992	401.1162	Villosolside	156599111
6	C_17_H_14_O_8_	0.992	347.079	Eupatolitin	5317291
7	C_17_H_24_O_11_	2.533	443.0963	Scandoside methyl ester	442433
8	C_17_H_24_O_10_	3.638	427.1032	Geniposide	107848
9	C_27_H_30_O_16_	3.739	611.1631	Rheinoside A	13888123
10	C_8_H_8_O_2_	4.241	137.0605	4-Hydroxyacetophenone	7469
11	C_5_H_7_NO_3_	4.342	147.0788	Pyroglutamic acid	7405
12	C_15_H_10_O_5_	4.442	271.0631	Aloe-emodin	10207
13	C_21_H_20_O_12_	4.542	465.105	Quercetin-3-glycoside	5280804
14	C_22_H_22_O_11_	4.576	463.1259	Cinnamoyl-glucogallin	53355598
15	C_10_H_8_O_4_	4.61	193.0513	Scopoletin	5280460
16	C_22_H_22_O_12_	5.146	479.1204	Isorhamnetin-3-glucoside	5318645
17	C_16_H_12_O_7_	5.146	317.0676	Capillarisin	5281342
18	C_28_H_32_O_15_	5.28	609.1833	Physciondiglucoside	73981703
19	C_15_H_10_O_6_	5.614	287.0573	Demethoxycapillarisin	5316511
20	C_11_H_10_O_4_	5.815	207.0665	Scoparone	8417
21	C_20_H_24_O_4_	7.591	329.1769	Crocetin	5281232
22	C_17_H_14_O_7_	8.998	331.0842	Eupalitin	5748611
23	C_17_H_14_O_6_	10.103	315.0879	Cirsimaritin	188323
24	C_16_H_12_O_5_	13.52	285.0773	Genkwanin	5281617
25	C_20_H_36_O_2_	16.669	326.3056	Mandenol	5282184
26	C_30_H_50_	22.13	411.3948	Supraene	638072
27	C_29_H_48_O	24.575	413.3792	Stigmasterol	5280794

**Table 2:** Primer sequences for Real-Time PCR.

No.	Target Genes	Primer Forward (5′->3′)	Primer Reverse (5′->3′)
1	*Cyp7a1*	GGGATTGCTGTGGTAGTGAGC	GGTATGGAATCAACCCGTTGTC
2	*Cyp8b1*	CCTCTGGACAAGGGTTTTGTG	GCACCGTGAAGACATCCCC
3	*Cyp27a1*	CCAGGCACAGGAGAGTACG	GGGCAAGTGCAGCACATAG
4	*Fxr*	GGCAGAATCTGGATTTGGAATCG	GCCCAGGTTGGAATAGTAAGACG
5	*Shp*	CTCATGGCCTCTACCCTCAA	GGTCACCTCAGCAAAAGCAT
6	*Bsep*	CAATGTTCAGTTCCTCCGTTCA	TCTCTTTGGTGTTGTCCCCATA
7	*Ntcp*	ACTGGCTTCCTGATGGGCTAC	GAGTTGGACGTTTTGGAATCCT
8	*Fgfr4*	CTGACTCGCAGACGACATGAG	AGGCCATGATCTTGAGATGAGA
9	*Mdr2*	CGGCGACTTTGAACTAGGCA	CAGAGTATCGGAACAGTGTCAAC
10	*Mrp2*	TCTTCGTCTCCTATGGTTTCCA	CGTGTGTTGAGTCGCTTGATT
11	*Mrp3*	CTGGGTCCCCTGCATCTAC	GCCGTCTTGAGCCTGGATAAC
12	*Mrp4*	GGCACTCCGGTTAAGTAACTC	TGTCACTTGGTCGAATTTGTTCA
13	*Oatp1a1*	GTGCATACCTAGCCAAATCACT	CCAGGCCCATAACCACACATC
14	*Oatp1a4*	ATAGCTTCAGGCGCATTTAC	TTCTCCATCATTCTGCATCG
15	*Oatp1b2*	GGGAACATGCTTCGTGGGATA	GGAGTTATGCGGACACTTCTC
16	*Asbt*	GTCTGTCCCCCAAATGCAACT	CACCCCATAGAAAACATCACCA
17	*Osta*	AGGCAGGACTCATATCAAACTTG	TGAGGGCTATGTCCACTGGG
18	*Ostβ*	AGATGCGGCTCCTTGGAATTA	TGGCTGCTTCTTTCGATTTCTG
19	*Zo-1*	GCCGCTAAGAGCACAGCAA	GCCCTCCTTTTAACACATCAGA
20	*Occludin*	TTGAAAGTCCACCTCCTTACAGA	CCGGATAAAAAGAGTACGCTGG
21	*Claudin-1*	GGGGACAACATCGTGACCG	AGGAGTCGAAGACTTTGCACT
22	*β-actin*	CGTTGACATCCGTAAAGACC	AACAGTCCGCCTAGAAGCAC

## Data Availability

The raw data supporting the conclusions of this article will be made available by the authors on request.

## Abbreviations

The following abbreviations are used in this manuscript:

AIF, all-ion fragmentation; ALP, alkaline phosphatase; ALT, alanine aminotransferase; ANIT, alpha-naphthyl isothiocyanate; ASBT, apical sodium-dependent bile acid transporter; AST, aspartate aminotransferase; BA, bile acid; BAs, bile acids; BCAAs, branched-chain amino acids; BSEP, bile salt export pump; CDCA, chenodeoxycholic acid; DBIL, direct bilirubin; DCA, deoxycholic acid; ESI, electrospray ionization; FDA, Food and Drug Administration; FGF15, fibroblast growth factor 15; FMT, fecal microbiota transplantation; FXR, farnesoid X receptor; GC-MS, gas chromatography–mass spectrometry; GCA, glycocholic acid; GDCA, glycodeoxycholic acid; H&E, hematoxylin and eosin; HDCA, hyodeoxycholic acid; ICP, intrahepatic cholestasis of pregnancy; LC-MS, liquid chromatography–mass spectrometry; LCA, lithocholic acid; MCA, muricholic acid; MDR2, multidrug resistance protein 2; MRP, multidrug resistance-associated protein; NTCP, sodium taurocholate cotransporting polypeptide; OCA, obeticholic acid; OST, organic solute transporter; PCA, principal component analysis; PCoA, principal coordinate analysis; PBC, primary biliary cholangitis; PFIC, progressive familial intrahepatic cholestasis; PLS-DA, partial least squares-discriminant analysis; PSC, primary sclerosing cholangitis; qPCR, quantitative polymerase chain reaction; RXR, retinoid X receptor; SCFA, short-chain fatty acid; SHP, small heterodimer partner; SRM, selective reaction monitoring; TBA, total bile acid; TBIL, total bilirubin; TCM, traditional Chinese medicine; TJ, tight junction; UDCA, ursodeoxycholic acid; UHPLC-MS, ultrahigh-performance liquid chromatography–mass spectrometry; YCHD, Yinchenhao decoction; ZO-1, zonula occludens-1.
